# Structural brain correlates of interpersonal violence: Systematic review and voxel-based meta-analysis of neuroimaging studies

**DOI:** 10.1016/j.pscychresns.2017.07.006

**Published:** 2017-09-30

**Authors:** Jelle Lamsma, Clare Mackay, Seena Fazel

**Affiliations:** Department of Psychiatry, University of Oxford, Warneford Hospital, Warneford Lane, OX3 7JX Oxford, United Kingdom

**Keywords:** Violence Grey matter volume MRI Prefrontal cortex Amygdala

## Abstract

Owing to inconsistent nomenclature and results, we have undertaken a label-based review and anatomical likelihood estimation (ALE) meta-analysis of studies measuring the quantitative association between regional grey matter (GM) volume and interpersonal violence. Following PRISMA guidelines, we identified studies by searching 3 online databases (Embase, Medline, PsycInfo) and reference lists. Thirty-five studies were included in the label-based review, providing information for 1288 participants and 86 brain regions. Per region, 0–57% of the results indicated significant reductions in GM volume, while 0–23% indicated significant increases. The only region for which more than half of all results indicated significant reductions was the parietal lobe. However, these results were dispersed across subregions. The ALE meta-analysis, which included 6 whole-brain voxel-based morphometry studies totaling 278 participants and reporting 144 foci, showed no significant clusters of reduced GM volume. No material differences were observed when excluding experiments using reactive violence as outcome or subjects diagnosed with psychopathy. Possible explanations for these findings are phenomenological and etiological heterogeneity, and insufficient power in the label-based review and ALE meta-analysis to detect small effects. We recommend that future studies distinguish between subtypes of interpersonal violence, and investigate mediation by underlying emotional and cognitive processes.

## Highlights

• We conducted a label-based review of 86 brain regions from 35 neuroimaging studies.
• In addition, we report a voxel-based meta-analysis of 144 foci from 6 studies.
• Regional grey matter volume was not consistently associated with violent outcomes.
• Future research should account for subtypes and situational aspects of violence.

## 1 Introduction

Over the last two decades, there have been numerous structural neuroimaging studies of interpersonal violence. However, the large number of different brain regions reported, variation in nomenclature and conflicting results have made interpretation difficult. Previous reviews have been non-systematic (e.g. Blair, 2010) or limited to small numbers of selected brain regions (e.g. Yang and Raine, 2009). It has also been common practice in reviews to conflate measures of violent behavior with indirect measures such as personality traits (e.g. poor impulse control, hostility) and psychiatric diagnoses (e.g. antisocial personality disorder [APD], psychopathy) (e.g. Brower and Price, 2001).

Understanding the neurobiological correlates of interpersonal violence is important for the development of: (1) interventions to prevent and reduce violence; (2) methods for screening and targeting individuals at risk for violence; (3) risk assessment tools informing involuntary admission, sentencing and release decisions; and (4) evaluation in criminal cases concerning the degree of a particular defendant's culpability and risk of future violence.

Therefore, we present a systematic label-based review (Radua and Mataix-Cols, 2012) of neuroimaging studies investigating the quantitative association between regional grey matter (GM) volume and interpersonal violence. We also performed an anatomical likelihood estimation (ALE) meta-analysis of voxel-based morphometry (VBM) studies examining volumetric reductions in regional GM.

## 2 Methods

This review was conducted in accordance with the Preferred Reporting Items for Systematic Reviews and Meta-Analyses (PRISMA) guidelines (Liberati et al., 2009).

### 2.1 Search strategy

We searched for studies indexed in the online databases Embase, Medline and PsycInfo from January 1990 to December 2014. Keywords were inclusive for violent behavior (“violen*”, “aggressi*”, “prison*”, “crim*”, “offen*”), structural neuroimaging techniques (“neuroimaging”, “brain imaging”, “computed tomography”, “CT”, “magnetic resonance imaging”, “MRI”, “diffusion tensor imaging”, “DTI”) and – to make the search more focused – brain regions that have usually received the most attention in neuroimaging studies of violent behavior (specifically “amygdala”, “prefrontal cortex”, “temporal cortex”). Additional studies were found by manually scanning the references of included studies and a number of recent review articles (e.g. Bufkin and Lutrell, 2005; Dolan, 2010; Fabian, 2010; Hoptman and Antonius, 2011; Patrick, 2008). Finally, we searched citations to included studies indexed in Google Scholar Citations from January 1990 to December 2014. Our search included grey literature (e.g. dissertations, conference papers, working papers). Authors were contacted if studies were unobtainable or additional information was required.

### 2.2 Study selection

Studies were eligible when meeting the following criteria: (1) the study contained primary data; (2) the study was available in the English language; (3) the study was conducted in or after 1990; (4) independent samples contained 10 or more participants; (5) all participants were aged 18 or older; (6) the study used in vivo neuroimaging by means of computed tomography or structural magnetic resonance imaging (including diffusion tensor imaging [DTI]); and (7) the study measured the quantitative association between violent behavior and at least one structural parameter (e.g. volume, fractional anisotropy) using between-group, correlation or regression analysis. We defined violent behavior as the intentional use of physical force to harm another person. To be included in the label-based review, the study had to provide sufficient information to code a result for at least one discrete brain region as negative, positive or non-significant. A negative result indicated a statistically significant association between violence and a reduction in GM volume, while a positive result indicated a statistically significant association between violence and an increase in GM volume. For ALE meta-analysis, we considered studies that conducted whole brain voxel-based analyses (VBAs) and reported coordinates for at least one peak voxel in either Montreal Neurological Institute or Talairach space.

We excluded: (1) samples that consisted of pedophilic offenders or participants with some form of brain lesion or malformation (e.g. cavum septum pellucidum), intellectual disability, epilepsy or a neurodegenerative disease (e.g. Huntington's disease, Alzheimer's disease); (2) analyses comparing qualitatively different types of violence (e.g. homicide versus rape); (3) psychiatric diagnoses and personality traits that are not defined by the actual display of violent behavior (e.g. psychopathy, impulsivity); and (4) instruments primarily designed to assess a person's inclination toward violent behavior (e.g. Buss-Perry Aggression Questionnaire, State-Trait Anger Expression Inventory).

### 2.3 Data extraction

For any combination of structural parameter and tissue class, we required a minimum of 5 experiments per: (1) brain region for label-based reviews; and (2) contrast of interest (i.e. reduction, increase) for ALE meta-analyses. Studies examining indices of white matter (WM) integrity (i.e. fractional anisotropy [*N* = 5], trace [*N* = 1], mean diffusivity [*N* = 1], radial diffusivity [*N* = 1]) with DTI, WM volume (*N* = 6) and cortical thickness (*N* = 2) contained insufficient experiments for label-based reviews and ALE meta-analyses. There was one VBM experiment of increases in GM volume of which peak-voxel coordinates were reported, precluding ALE meta-analysis. Consequently, we performed: (1) a label-based review of studies examining GM volume; and (2) an ALE meta-analysis of VBM studies examining reductions in GM volume.

The first author assessed suitability of studies for inclusion and used a standardized form to collect information from each study such as design, country, sample size, psychiatric morbidity and definition of violence. Any uncertainties were resolved by discussion with the other authors. A research assistant checked data extraction accuracy of 10 randomly selected studies; correspondence was more than 99%.

To facilitate the exploration and interpretation of results, we divided the brain into the following regions of interests (ROIs): frontal lobe; prefrontal cortex; dorsolateral prefrontal cortex (dlPFC); ventrolateral prefrontal cortex; medial prefrontal cortex; orbitofrontal cortex (OFC); anterior cingulate cortex (aCC); posterior frontal cortex; temporal lobe, lateral temporal lobe; medial temporal lobe; amygdala, hippocampus; polar temporal lobe; parietal lobe; postcentral gyrus; superior parietal lobule; inferior parietal lobule; occipital lobe; lateral occipital lobe; medial occipital lobe; cingulate cortex; posterior cingulate cortex; fusiform gyrus; temporal fusiform gyrus; occipital fusiform gyrus; striatum; and other subcortical structures (e.g. hypothalamus, cerebellum). Additional information on data extraction can be found in the online supplement.

### 2.4 Data analysis

#### 2.4.1 Label-based review

Weighting by sample size, we calculated the percentages of negative, positive and non-significant results reported for each brain region. Statistical significance was determined with: (1) an α level of 0.05 (two-tailed) for results of ROI analyses; and (2) the thresholding criteria applied by the study authors for results of VBM analyses. We rejected the null hypothesis if more than 50% of the results were all either negative or positive.

#### 2.4.2 ALE meta-analysis

ALE meta-analysis was carried out in GingerALE 2.3.6 (brainmap.org/ale). We used the non-additive algorithm (Turkeltaub et al., 2012) to minimize within-experiment effects. Inference was made at cluster-level (*p* < 0.05, 1000 permutations) with an uncorrected voxel-wise *p*-value of 0.005. Cluster-level inference has been shown to provide a better balance between sensitivity and specificity compared with other methods to correct for multiple comparisons currently available in GingerALE (Eickhoff et al., 2012). The α levels are in line with those used in previous ALE meta-analyses (e.g. Barron et al., 2013; Fusar-Poli et al., 2013).

#### 2.4.3 Subgroup analyses

It has been theorized that the neurobiological correlates of violent behavior differ between reactive *vs* proactive (Rosell and Siever, 2015) and adolescence-limited *vs* life course-persistent (Moffitt and Caspi, 2001) subtypes. While we planned subgroup analyses of these subtypes for both the label-based review and ALE meta-analysis, only sufficient experiments were available to add a subgroup analysis of reactive violence to the label-based review.

#### 2.4.4 Sensitivity analyses

To determine the robustness of the findings, we repeated both the label-based review and ALE meta-analysis after separately excluding experiments with: (1) reactive violence as outcome; and (2) samples that consisted of subjects diagnosed with psychopathy (Anderson and Kiehl, 2014).

## 3 Results

Fig. S1 shows a flow diagram of the search process.

### 3.1 Label-based review

There were 35 studies that met inclusion criteria for the label-based review. These studies contained a total of 1288 participants with a mean age of 33 years (range = 20 – 48 years). Most participants were male (*n* = 1066, 83%) and nearly half (*n* = 575, 45%) were diagnosed with one or more of the following (classes of) psychiatric disorders: axis I disorder (*n* = 390; 30%); personality disorder (*n* = 276; 21%); schizophrenia or schizoaffective disorder (*n* = 244; 19%); APD (*n* = 132; 10%); borderline personality disorder (BPD, *n* = 115; 9%); alcohol abuse or dependence (*n* = 105; 8%); psychopathy (*n* = 68; 5%); intermittent explosive disorder (IED, *n* = 29; 2%); and dissocial personality disorder (DPD, *n* = 26; 2%). For additional characteristics of studies included in the label-based review, see Table S1.

**Fig. 1:**
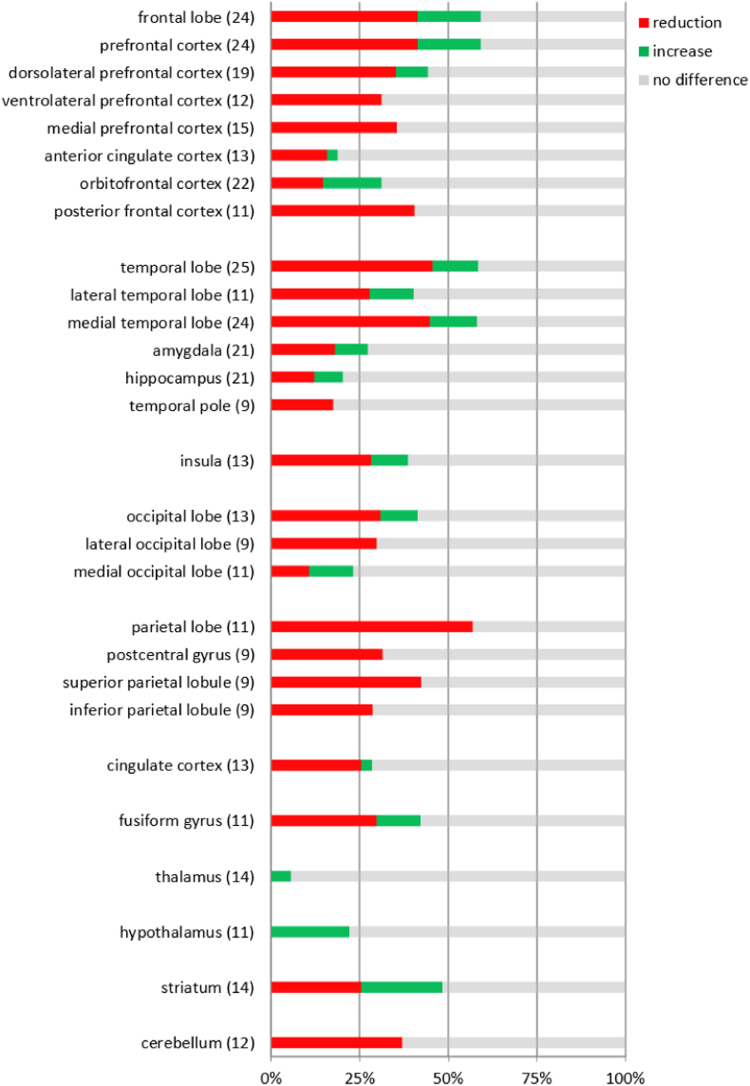
Percentages of neuroimaging experiments indicating whether interpersonal violence was associated with a reduction, increase or no difference in grey matter volume in regions of interest (ROI). Experiments were weighted by sample size. For each ROI, the total number of results is given between parentheses.

Fig. S2 shows the percentages of negative, positive and non-significant results reported for all brain regions (*k* = 86). Fig. 1 contains the same information, but only for our ROIs (*k* = 28). The total number of results available for any one region ranged from 9 to 25. For almost all regions, most results were non-significant. The parietal lobe (57%; 7 out of 11) was the only region for which more than half of the results were negative, indicating that violence was significantly associated with reduced GM volume. For the remaining regions, negative results accounted for 46% or less of the total number of results. Percentages of positive results, reflecting a significant association between violent behavior and increased GM volume, varied between 0% and 23%.

#### 3.1.1 Reactive violence

Seven studies were included in the subgroup analyses of reactive violence (Table S1). These studies contained a total of 389 predominantly male (*n* = 362, 93%) participants, who had a mean age of 26 years (range = 20 – 40 years). Approximately 13% (*n* = 53) of the participants were diagnosed with either IED (*n* = 29, 7%) or alcohol dependence (*n* = 24, 6%).

The percentages of negative and positive results for all brain regions (*k* = 5) did not exceed 37% and 18%, respectively (Fig. S3).

#### 3.1.2 Sensitivity analyses

We observed no material differences in overall findings when repeating the label-based review after excluding experiments with reactive violence as outcome (Fig. S4) or those with samples that consisted of subjects diagnosed with psychopathy (Fig. S5).

### 3.2 ALE meta-analysis

Six VBM studies that examined reductions in regional GM volume met inclusion criteria for the ALE meta-analysis (Table S2). These studies reported 144 foci for 7 group comparisons of 13 independent samples, totaling 278 participants. The mean age was 34 years (range = 26 – 41 years), and less than a third (*n* = 90) of the participants were diagnosed with one or more of the following psychiatric disorders: ADP (*n* = 55, 20%); psychopathy (*n* = 51, 18%); BPD (*n* = 13, <1%); alcohol abuse or dependence (*n* = 26, 1%); and DPD (*n* = 26, 1%).

The ALE meta-analysis showed no significant clusters of reduced GM volume in violent subjects compared to non-violent subjects.

#### 3.2.1 Sensitivity analyses

No significant clusters of reduced GM volume in comparisons between violent and non-violent subjects were found in either sensitivity analysis.

## 4 Discussion

To our knowledge, this is the most comprehensive review to date of neuroimaging studies investigating the association between regional GM volume and interpersonal violence. Thirty-five studies with a total of 1288 participants were included in a label-based review of 86 brain regions. In subgroup analyses of reactive violence, 7 studies with a total of 389 participants and 5 brain regions were included. We also performed an ALE meta-analysis of 6 VBM studies with a total of 278 participants. Neither the label-based review nor the ALE meta-analysis showed consistent associations between regional GM volume and interpersonal violence.

Our findings suggest that, in the absence of gross pathology, GM volume of discrete brain regions is not a reliable neuroimaging marker of violence. This also applies to regions implicated by current theories, including the OFC (Davidson et al., 2000b), dlPFC (Davidson et al., 2000a), aCC (Siever, 2008), amygdala (DeLisi et al., 2009) and hippocampus (Yang et al., 2008). While most results for the parietal lobe in the label-based review indicated statistically significant reductions in GM volume, interpretation is hampered by the anatomo-functional heterogeneity of this region and the internal inconsistency of the results.

We propose phenomenological and etiological heterogeneity as the primary explanation for our findings. The construct of interpersonal violence encompasses a wide range of behaviors that arise from multifarious interactions of environment with emotional and cognitive processes (e.g. fear conditioning, impulse control, moral reasoning) mediated by different, interconnected brain regions (Raine, 2008). It seems unlikely that all these behaviors are captured by a single neuroimaging marker. Relatedly, the failure in most studies to distinguish between subtypes (e.g. reactive *vs* proactive, adolescence-limited *vs* life course-persistent) and control for situational aspects (e.g. acute intoxication, peer group pressure) of violence may have attenuated or even obscured associations. Although the findings from the subgroup analysis suggest that reactive violence is no more consistent as outcome than generic violence, the small numbers of results and brain regions included warrant cautious interpretation. To end, all studies relied on retrospective measurement or had potentially long time lags between scan and violent behavior. This, too, may have diminished the ability to detect associations.

A number of important limitations to this review should be discussed. First, power is not aggregated across experiments in label-based review or ALE meta-analysis. This is compounded by the often small samples used in experiments. As a consequence, some true effects may have been missed. We decided against label-based meta-analysis of effect sizes for the following reasons: (1) as VBM studies only report effect sizes for significant results, combining VBM and ROI studies in the same meta-analysis could lead to biased estimation of the mean effect size; (2) preliminary examination demonstrated high levels of statistical heterogeneity; and (3) we considered the numbers of available effect sizes combined with the small sample sizes used in most ROI experiments insufficient to conduct subgroup or meta-regression analyses. Second, inclusion of subjects who were diagnosed with psychiatric disorders (e.g. schizophrenia (Honea et al., 2005), BPD (Lis et al., 2007)) and, in some instances, treated with medication (e.g. antipsychotics (Lieberman et al., 2005)) may have contributed to inconsistencies in results reported for some brain regions. Finally, we conducted label-based reviews and ALE meta-analyses if at least 5 experiments were available for the same brain region and contrast of interest, respectively. While deemed necessary to improve the validity of the findings, this approach excluded potentially relevant structural parameters (e.g. fractional anisotropy, cortical thickness) and tissue classes (e.g. WM, cerebrospinal fluid).

Several implications for future research arise from this review. First, phenomenological and etiological heterogeneity of violent behavior could be reduced by distinguishing between subtypes, control for situational aspects, and consideration of the emotional and cognitive processes that lie on intermediate pathways. Second, possible abnormalities in violent individuals are most rigorously investigated with whole-brain analyses of multiple combinations of structural parameter and tissue class. Finally, prospective designs with short intervals between waves (weeks or months rather than years) may improve the reliability and validity of results.

## Contributors

JL and SF conceived and designed the analyses; JL collected the data and performed the analyses; JL, CM and SF analyzed the data; JL drafted the manuscript, and CM and SF critically reviewed and revised it.
