# Supplementary material for: Structural brain correlates of interpersonal violence: Systematic review and voxel-based meta-analysis of neuroimaging studies
Source: Psychiatry Res. 2017 Sep 30;267:69–73. doi: 10.1016/j.pscychresns.2017.07.006 (PMC5670119; doi:10.1016/j.pscychresns.2017.07.006)
Supplement: Supplementary file 1 — Supplementary material [file mmc1.docx]

**Further details regarding data extraction**

*Parcellation scheme*

We used the parcellation scheme described below to categorize results. The cerebral cortex was divided into frontal, temporal, parietal, occipital and insular lobes. The frontal lobe was subdivided in the prefrontal cortex (PFC) and posterior frontal cortex. We used the parcellation of the PFC proposed by Yang and Raine (2009), discerning the dorsolateral prefrontal cortex (dlPFC), ventrolateral prefrontal cortex (vlPFC), medial prefrontal cortex (mPFC), orbitofrontal cortex (OFC) and anterior cingulate cortex (aCC). The definitions of Yang and Raine (2009) served to delineate the dlPFC (Brodmann Areas [BAs] 8, 9, 10 and 46), vlPFC (BAs 44 and 45), mPFC (medial sections of BAs 8, 9, 10, 11 and 12) and OFC (BAs 11, 12 and 47). For the aCC (BAs 24, 25, 32 and 33), we integrated the definitions used by Hoffstaedter et al. (2014) and Kozslovskiy et al. (2012). The temporal lobe was subdivided in lateral, medial and polar aspects (Kiernan, 2012). The medial temporal lobe included the amygdala and hippocampus. Results for these structures are also presented separately given their hypothesized importance for violent behavior. The parietal lobe was subdivided in the postcentral gyrus, superior parietal lobule and inferior parietal lobule. The occipital lobe was subdivided in lateral and medial aspects. Results were available for two structures that encompass parts of more than one lobe: the cingulate cortex and the fusiform gyrus. The cingulate cortex was divided in the anterior cingulate cortex (aCC) and posterior cingulate cortex (pCC). The pCC, part of the parietal lobe, was defined as BAs 23 and 31 (Hoffstaedter et al., 2014; Kozslovskiy et al., 2012). The fusiform gyrus was divided in temporal (BAs 20, 36 and 37) and occipital (BAs 18 and 19) aspects (Kiernan, 2012; Rademacher et al., 1992). Results are also presented for the entire cingulate cortex and fusiform gyrus. The caudate nucleus, putamen, nucleus accumbens and subdivisions containing different parts of these regions (e.g. lentiform nucleus, ventral striatum) were grouped together as the striatum (Nolte, 2009). Other subcortical structures, such as the hypothalamus and cerebellum, were considered separately. A result recorded for a particular brain region was also recorded for one or more brain regions of which the former is part (e.g. a negative result for the parahippocampal gyrus also counted as a negative result for both the medial temporal lobe and the temporal lobe).

*Derivation of labels*

We adopted the labels used by the authors in each study to identify brain regions. One label was agreed upon if different labels were used across studies for essentially the same brain region (e.g. limbic striatum and ventral striatum). Results of studies that used different labels for the ROIs described above (e.g. lateral prefrontal cortex instead of dlPFC) were classified based on the information they provided (i.e. anatomical landmarks, BAs, peak voxel coordinates). The results for two ROIs examined in a study using an ROI approach (Barkataki et al., 2006) were not classified, because they comprised large parts of two or more ROIs examined for the purpose of this review. To classify results of voxel-based morphometry (VBM) studies that used two labels for the same set of peak voxel coordinates, we used either: (1) the label that was most specific (e.g. precuneus instead of parietal lobe); or (2), if neither label was more specific than the other (e.g. amygdala and hippocampus), the peak voxel coordinates. Peak voxel coordinates were also used to classify results in cases where VBM studies used labels that contained parts of more than one ROI (e.g. paracentral gyrus). Alternative labels for peak voxel coordinates were obtained with the Automated Anatomical Labeling atlas (Tzourio-Mazoyer et al., 2002) or BA atlas implemented in MRIcron (Rorden et al., 2007). One result in Bertsch et al. (2013) was not classified, because the location of the corresponding peak voxel coordinates could not reliably be determined with either atlas.

*VBM and ROI analyses*

If both whole-brain VBM and ROI analyses were done in the same sample, we included the results of the former given its ability to detect highly localized tissue differences across the whole brain with minimal user bias.

Volumetric changes are introduced when registering an individual’s image to a standard brain template. In VBM, original volumes can be preserved by modulating the spatially normalized images. Modulation entails multiplying each voxel’s probability of belonging to a specific tissue class by a scaling factor proportionate to the volumetric change introduced by the spatial normalization procedure (Radua et al., 2014). If both modulated and unmodulated voxel-wise analyses were conducted in the same sample, the results of the former were included to optimize comparison among studies. We selected the results from the voxel-wise analysis that applied the most stringent correction for multiple comparisons. The latter two criteria also applied to VBM studies included in the Anatomical Likelihood Estimation meta-analysis.

Results of ROI analyses that did not involve either segmentation or tracing of GM were only included if they involved brain regions that are wholly or largely composed of GM (e.g. prefrontal cortex, amygdala).

*Overlapping samples*

If more than one result was available for the same brain region in overlapping samples of unequal size, we included the result of the analysis that used the largest sample. If two or more different studies provided results for the same brain region in overlapping samples of equal size, we included the result of either: (1) the study that provided the most accurate data for the calculation of group differences (Barkataki, et al., 2006); (2) the study conducting the analysis that adjusted for the maximum number of control variables (Barkataki et al., 2006; Gansler et al., 2009; Gilliam, 2014); or (3), *ceteris paribus*, the most recent study (Kumari et al., 2013). If the same study provided more than one result for the same brain region in overlapping samples of equal size, we included either: (1) the result of the analysis that adjusted for the maximum number of control variables (Barkataki et al., 2006; Dolan et al., 2002a; Gansler et al., 2009; Hoptman et al., 2006; Laakso et al., 2002; Yang et al., 2010; Zhang et al., 2013); (2), if the number of control variables was equal, the result of the analysis with the control variable(s) most often used in other studies (Gansler et al., 2009); or (3), *ceteris paribus*, one result per category (e.g. negative) for which results were available (Bertsch et al., 2013; Bobes et al., 2013; Gregory et al., 2012; Tiihonen et al., 2008). We were unable to ascertain the presence of overlap between the samples reported on in 4 papers from the same research group (Frankle et al., 2005; New et al., 2007; Rosell et al., 2010; van de Giessen et al., 2014). These samples were treated as overlapping.

Unless a study reported only bilateral results, we used the following coding scheme: (1) a non-significant result was recorded if the results for both hemispheres were non-significant; (2) a negative or positive significant result was recorded if the results for both hemispheres were significant in a negative or positive direction, respectively; (3) a negative or positive significant result was recorded if the result for one hemisphere was either non-significant or not reported on and the result for the other hemisphere was significant in a negative or positive direction, respectively.

An analogous approach was used to combine results for a main region (e.g. amygdala) if results were available for one or more of its subregions (e.g. ventral amygdala) in overlapping samples: (1) a non-significant result was recorded if the result for the main region or all results for its subregions were non-significant; (2) a negative or positive significant result was recorded if for the main region or at least one of its subregions a negative or positive result was reported, respectively; (3) results belonging to the same category (e.g. negative) counted as one result.

*Multiple appropriate violent outcomes*

In instances where the same study used more than one appropriate instrument to measure violent behavior, we included the results of the analysis based on the instrument that either: (1) measured the most severe type of violent behavior (Spoletini et al., 2011); (2) was most often used in other studies (Hoptman et al., 2005); or (3) measured violent behavior across the lifespan instead of that up to the age of 15 (Schiffer et al., 2013). Using the same instrument, one study (Hoptman et al., 2006) conducted both correlation and between-group analyses. As it provided a more detailed assessment by taking into account both the frequency and severity of violent behavior, we included the result of the correlation analysis. From Pardini et al. (2014), we included the results of the analyses that combined self-report and criminal records for the ascertainment of violence.

*Psychiatric morbidity*

Psychiatric morbidity was recorded if all participants in one sample were diagnosed with the same psychiatric disorder or class of psychiatric disorders (e.g. axis I, personality disorder). Where possible, we chose appropriate psychiatric controls over healthy controls.

*Analyses of variance (ANOVAs) and multivariate analyses of variance (MANOVAs)*

Two-tailed *t*-tests with an α level of 0.05 were used to compare a group of violent participants with a group of non-violent participants with respect to one brain region in cases where studies provided sample sizes, means and standard deviations for both groups and: (1) used ANOVA with three or more groups, including at least one group of violent participants and at least one group of non-violent participants, or two or more brain regions as independent variables; or (2) used MANOVA with two or more groups, including at least one group of violent participants and at least one group of non-violent participants, or two or more brain regions as independent variables, and two or more brain regions as dependent variables. Results of ANOVAs with two groups, one group of violent participants and one group of non-violent participants, and one brain region as independent variable were extracted as normal.

*Excluded experiments*

Since our aim was to analyze the direct relationship between violent behavior and the structural properties of discrete brain regions, and also to ensure comparability of results between studies, we decided to exclude: (1) interaction effects; (2) results of analyses that statistically controlled for the effect of one or more brain regions other than the brain region of interest; (3) ratios between different brain regions; and (4) measures of hemispheric asymmetry.


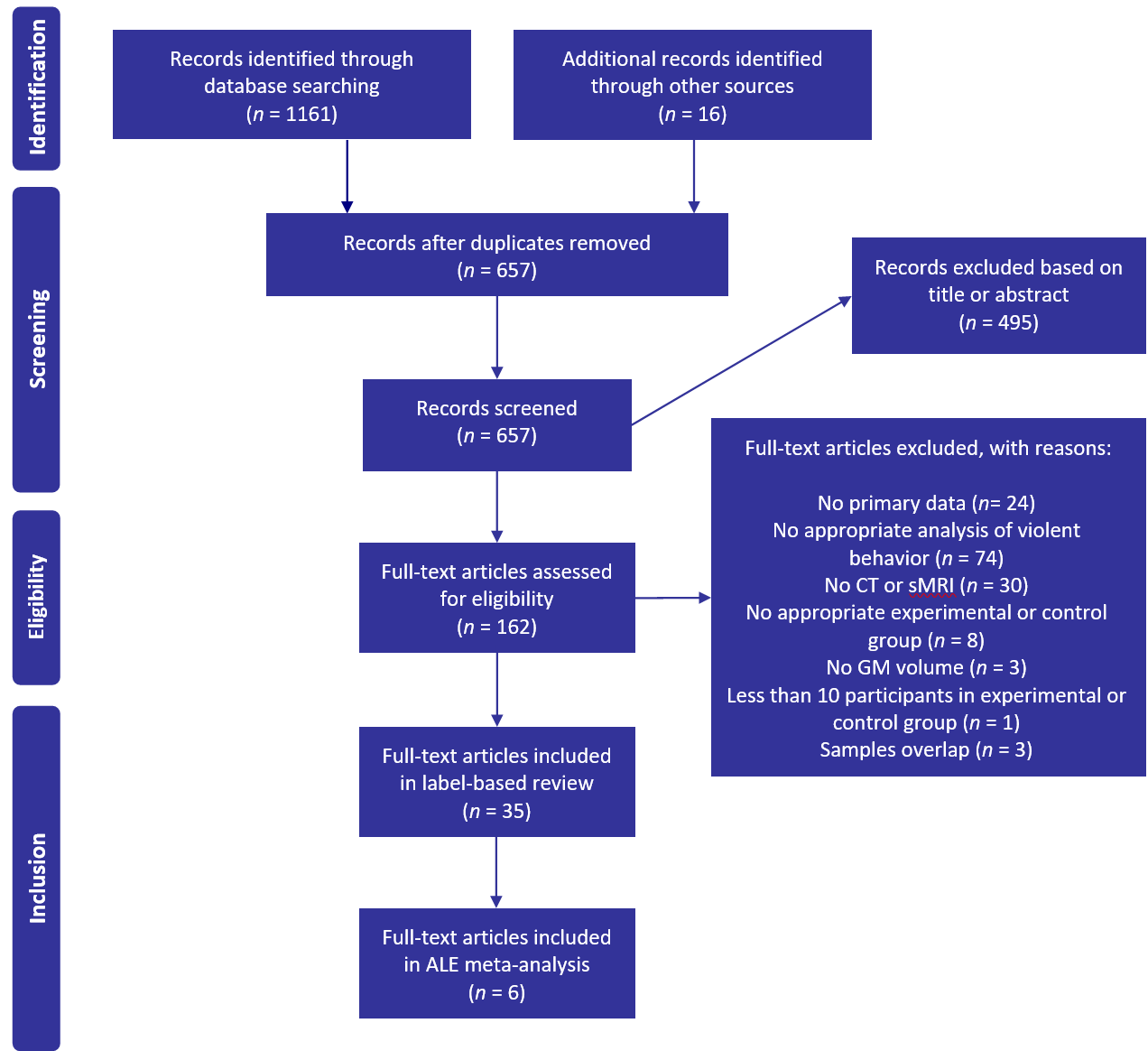


**Figure S1.** Preferred Reporting Items for Systematic Reviews and Meta-Analyses (PRISMA) diagram showing the search strategy used to identify the studies included in the label-based review and Anatomical Likelihood Estimation (ALE) meta-analysis. CT = computed tomography; GM = grey matter; sMRI = structural magnetic resonance imaging. Adapted from Moher et al. (2009).

**Table S1.** Characteristics of studies (*N* = 35) included in the label-based review.

Experimental group Control group

Post-

Method of Psychiatric Diagnostic Type of processing

Reference Country *N* (male *n*) Age^1^ recruitment morbidity criteria *N* (male *n*) Age^1^ control group technique Definition of violence

Barkataki et al. (2006) [1] U.K. 13 (13) 34.46 Institutional Sz DSM-IV 15 (15) 34.47 Psychiatric ROI A minimum score of 4 on the GRS, indicating a

(near) fatal act of violence against another person

Barkataki et al. (2006) [2] U.K. 13 (13) 31.62 Institutional APD DSM-IV 15 (15) 32.13 Healthy ROI A minimum score of 4 on the GRS, indicating a

(near) fatal act of violence against another person

Bertsch et al. (2013) [1] Germany 12 (12) 27.30 Institutional APD, DSM-IV, 14 (14) 26.10 Healthy VBM Conviction for crime that causes severe injury, Psych PCL-R such as murder, manslaughter, robbery or rape

Bertsch et al. (2013) [2] Germany 13 (13) 28.90 Institutional APD, DSM-IV 14 (14) 26.10 Healthy VBM Conviction for crime that causes severe injury,

BPD such as murder, manslaughter, robbery or rape

Bobes et al. (2013)^2^ Mexico 25 (25) 30.63 Community None na 29 (29) 28.93 na VBM A minimum score of 9 on the Reactive Aggression

subscale of the RPAQ

Contreras-Rodríguez Spain 22 (22) 39.80 Institutional Psych PCL-R 22 (22) 40.60 Healthy VBM Severe criminal offense history (i.e. murder and

et al. (2014) armed robbery)

Dolan et al. (2002a) U.K. 17 (17) ns Institutional Psych, DSM-III-R, 12 (12) ns Healthy ROI Conviction for a violent offense, mostly murder

PD SHAPS

Dolan et al. (2002b) U.K. 43 (43) ns Mixed None na na na na ROI Total score on the BGA

Frankle et al. (2005)^2^ U.S.A. 10 (5) 35.00 Community IED-R, Coc, 10(5) 34.00 Healthy ROI Meeting diagnostic criteria for IED-R

PD DSM-IV

Gansler et al. (2009) [1] U.S.A. 41 (36) 40.12 Mixed Axis I DSM-IV na na na ROI Score on the subscale for verbal and physical

aggression of the LHA-R

Gansler et al. (2009) [2] U.S.A. 19 (18) 40.94 Community None na na na na ROI Score on the subscale for verbal and physical

aggression of the LHA-R

(Table continues)

**Table S1.** (Continued)

Experimental group Control group

Post-

Type of Psychiatric Diagnostic Type of processing

Reference Country *N* (male *n*) Age^1^ recruitment morbidity criteria *N* (male *n*) Age^1^ control group technique Definition of violence

Gansler et al. (2011) U.S.A. 36 (36) 39.47 Mixed Axis I DSM-IV na na na ROI Score on the Aggression subscale of the LHA-R

Gilliam (2014)^2^ U.S.A. 169 (169) 20.00 Community None na na na na ROI Frequency of reactive aggressive acts within

the past year, measured with a subset of items

from the SRD

Gilliam et al. (2014) U.S.A. 169 (169) 20.00 Community None na na na na ROI Frequency of reactive aggressive acts within the

past year, measured with a subset of items from

the SRD

Gregory et al. (2012) [1] U.K. 17 (17) 38.90 Institutional APD, DSM-IV, 22 (22)^3^ 32.40 Healthy VBM History of one or more convictions for violent

Psych PCL-R crime (i.e. murder, attempted murder, rape and

grievous bodily harm)

Gregory et al. (2012) [2] U.K. 27 (27)^4^ 36.10 Institutional APD DSM-IV 22 (22)^3^ 32.40 Healthy VBM History of one or more convictions for violent

crime (i.e. murder, attempted murder, rape and

grievous bodily harm)

Gopal et al. (2013) U.S.A. 41 (36) 40.00 Mixed Axis I DSM-IV na na na ROI Score on the subscale for verbal and physical

aggression of the LHA-R

Hoptman et al. (2005) U.S.A. 49 (43) 41.50 Institutional Sz/SAD DSM-IV na na na ROI Total weighted score on the OAS, reflecting the

seriousness and frequency of violent incidents

during the study period

Hoptman et al. (2006) U.S.A. 49 (43) 41.50 Institutional Sz/SAD DSM-IV na na na ROI Total weighted score on the OAS, reflecting the

seriousness and frequency of violent incidents

during the study period

Kumari et al. (2009) U.K. 10 (10) 35.00 Institutional Sz DSM-IV 14 (14) 33.80 Psychiatric ROI A score of 5 or above on the GRS, indicating at

least one (near) fatal act of violence against the

victim

(Table continues)

**Table S1.** (Continued)

Experimental group Control group

Post-

Type of Psychiatric Diagnostic Type of processing

Reference Country *N* (male *n*) Age^1^ recruitment morbidity criteria *N* (male *n*) Age^1^ control group technique Definition of violence

Kumari et al. (2013) [1] U.K. 13 (13) 34.46 Institutional Sz DSM-IV 15 (15) 34.47 Psychiatric ROI A minimum score of 4 on the GRS, indicating a

(near) fatal act of violence against another person

Kumari et al. (2013) [2] U.K. 13 (13) 31.61 Institutional APD DSM-IV 15 (15) 32.13 Healthy ROI A minimum score of 4 on the GRS, indicating a

(near) fatal act of violence against another person

Kumari et al. (2014) U.K. 57 (57) 33.07 Mixed None na na na na ROI Score on the GRS

Laakso et al. (2000) Finland 19 (19) 30.00 Institutional APD, Clo, 17 (17) 48.00 Psychiatric^5^ ROI Charged with a violent offense (i.e. murder, Alc-II DSM-IV, attempted murder, manslaughter, assisting ICD-10 manslaughter, armed robbery, assault and

aggravated assault)

Laakso et al. (2002) Finland 24 (24) 31.00 Institutional APD, Clo, 33 (33) 34.00 Healthy ROI Charged with a violent offense (i.e. murder, Alc-II DSM-IV, attempted murder, manslaughter, assisting ICD-10 manslaughter, armed robbery, assault and

aggravated assault)

Matthies et al. (2012) Germany 20 (0)^6^ 27.20 Community None na na na na ROI Score on the subscale for verbal and physical

aggression of the LHA-R

New et al. (2007)^2^ U.S.A. 26 (19) 33.97 ns IED-R, Coc, 24 (15)^7^ 32.56 Healthy ROI Meeting diagnostic criteria for IED-R

BPD DSM-IV

Pardini et al. (2014) [I] U.S.A. 21 (21) 26.00 Community None na 35 (35) 26.00 na ROI Reporting an act of violence on the SRD and/or

being charged with a violent crime within 3 years

after the scan

(Table continues)

**Table S1.** (Continued)

Experimental group Control group

Post-

Type of Psychiatric Diagnostic Type of processing

Reference Country *N* (male *n*) Age^1^ recruitment morbidity criteria *N* (male *n*) Age^1^ control group technique Definition of violence

Pardini et al. (2014) [II]^2^ U.S.A. 56 (56) 26.00 Community None na na na na ROI Score on the subscale for impulsive aggression of

the IAR

Puri et al. (2008) U.K. 13 (12) 40.40 Institutional Sz DSM-IV 13 (10) 32.60 Psychiatric VBM Violent offending (i.e. homicide, attempted

homicide and wounding with intent to cause

grievous bodily harm) prior to admission,

considered by court-accepted expert opinion to

be a direct result of schizophrenia

Rosell et al. (2010) [1]^2^ U.S.A. 14 (10) 36.75 Community IED-IR+A2, DSM-IV, 25 (15) 32.86 Healthy ROI Meeting diagnostic criteria for IED-IR

PD McC

Rosell et al. (2010) [2]^2^ U.S.A. 15 (12) 36.40 Community IED-IR-A2, DSM-IV, 25 (15) 32.86 Healthy ROI Meeting diagnostic criteria for IED-IR

PD McC

Schiffer et al. (2011) Germany 24 (24) 36.90 Institutional None na 27 (27) 36.99 na VBM Conviction for a violent offense

Schiffer et al. (2013) [1] Germany 50 (50) 35.97 Mixed Sz DSM-IV na na na ROI Composite score on the fighting and assault items

of the LHA-R

Schiffer et al. (2013) [2] Germany 52 (52) 34.56 Mixed None na na na na ROI Composite score on the fighting and assault items

of the LHA-R

Spoletini et al. (2011) Italy 50 (29) 40.67 Community Sz DSM-IV-TR na na na ROI Score on the Physical Aggression subscale of the

OAS-M

Soloff et al. (2014) [1] U.S.A. 16 (5) 36.10 Community BPD DIB, DIB-R na na na SVC Total score on the BGA

ICD-10

Soloff et al. (2014) [2] U.S.A. 35 (5) 27.40 Community BPD DIB, DIB-R na na na SVC Total score on the BGA ICD-10

(Table continues)

**Table S1.** (Continued)

Experimental group Control group

Post-

Type of Psychiatric Diagnostic Type of processing

Reference Country *N* (male *n*) Age^1^ recruitment morbidity criteria *N* (male *n*) Age^1^ control group technique Definition of violence

Tiihonen et al. (2008) Finland 26 (26) 32.50 Institutional Alc-II, Clo, 25 (25) 34.60 Healthy VBM Charged with a violent offense (i.e. murder,

APD, DSM-IV, attempted murder, manslaughter, attempted

DPD ICD-10 manslaughter, assisting manslaughter, assault,

armed robbery)

Van de Giessen et al. U.S.A. 29 (11) 39.70 ns IED-IR, DSM-IV, 30 (21) 35.50 Healthy ROI Diagnostic criteria for IED-IR

(2014)^2^ PD McC

Yang et al. (2010) [1] China 22 (3) 34.68 Institutional Sz CCMD-3, 19 (3) 33.11 Psychiatric ROI Accused of homicide^8^

DSM-IV

Yang et al. (2010) [2] China 18 (2) 31.39 Institutional None na 32 (4) 32.03 na ROI Accused of homicide^8^

Zetzsche et al. (2007) Germany 25 (0) 26.10 Institutional BPD DIB-R, na na na ROI Total score on the BGA

DSM-IV

Zetzsche et al. (2008) Germany 25 (0) 26.70 Institutional BPD DIB-R, na na na ROI Total score on the BGA

DSM-IV

Zhang et al. (2013)^2^ U.S.A. 51 (51) 31.86 Community None na na na na ROI Score on the subscale for physical aggression of

the CTS

Zhang et al. (2013) [a]^2^ U.S.A. 24 (24) 35.00 Community AD DSM-IV 14 (14) 38.90 Psychiatric ROI At least two acts of physical aggression (i.e. hitting

punching, aggressive pushing, shoving, choking or

using a weapon) toward a spouse or significant

other in the past year. These acts were not

premeditated and typically associated with

affective symptoms (e.g. palpitations, tremors)

Bracketed numbers indicate separate analyses within the same study involving different experimental groups; bracketed Latin numerals indicate separate analyses within the same study involving the same experimental and control groups; bracketed lower-case letters indicate separate analyses within the same study involving subsamples. AD = alcohol dependence; Alc-II = type II alcoholism; APD = antisocial personality disorder; BGA = Brown–Goodwin Assessment for Lifetime History of Aggression (Brown et al., 1979); BPD = borderline personality disorder; CCMD-3 = Chinese Classification of Mental Disorders, Third Edition (Chinese Society of Psychiatry, 2001); Clo = Cloninger et al. (1981) and Cloninger (1987a; 1987b); Coc = Coccaro et al., 1998; CTS = Conflict Tactics Scale (Straus, 1979); DIB = Diagnostic Interview for Borderlines (Gunderson et al., 1981); DIB-R = Diagnostic Interview for Borderlines-Revised (Zanarini et al., 1989); DPD = dissocial personality disorder; DSM-III-R = Diagnostic and Statistical Manual of Mental Disorders, Third Edition, Revised (American Psychiatric Association, 1987); DSM-IV = Diagnostic and Statistical Manual of Mental Disorders, Fourth Edition (American Psychiatric Association, 1994); DSM-IV-TR = Diagnostic and Statistical Manual of Mental Disorders, Fourth Edition, Text Revision (American Psychiatric Association, 2000); GRS = Gunn and Robertson Scale (Gunn and Robertson, 1976); IAR = Impulsive-Premeditated Aggression Scale (Stanford et al., 2003); ICD-10 = International Classification of Diseases, 10th Revision (World Health Organization, 1992); IED-IR = intermittent explosive disorder-integrated research; IED-IR+A2 = intermittent explosive disorder-integrated research, meeting criterion A2 (i.e. three or more acts of physical assault against other people or destruction of property over a 1-year period) for the past year; IED-IR-A2 = intermittent explosive disorder-integrated research, not meeting criterion A2 (i.e. three or more acts of physical assault against other people or destruction of property over a 1-year period) for the past year; IED-R = intermittent explosive disorder-revised; LHA-R = Life History of Aggression-Revised (Coccaro et al., 1997); McC = McCloskey et al., 2006; na = not applicable; ns = not specified; OAS = Overt Aggression Scale (Yudofsky et al., 1986); OAS-M = Overt Aggression Scale-Modified (Kay et al., 1988); PD = personality disorder; PCL-R = Psychopathy Checklist-Revised (Hare, 2003); Psych = psychopathy; RPAQ = Reactive and Proactive Aggression Questionnaire (Raine et al., 2006); ROI = region of interest analysis; SAD = schizoaffective disorder; SHAPS = Special Hospital Assessment of Personality and Socialization (Blackburn, 1982); SRD = Self-Report of Delinquency (Elliot et al., 1985); SVC = small volume corrected voxel-based morphometry analysis; Sz = schizophrenia; U.K. = United Kingdom; U.S.A. = United States of America; VBM = whole-brain voxel-based morphometry analysis

^1^ Mean age in years

^2^ Study included in the subgroup analysis of reactive violence

^3^ Due to technical difficulties, the scans of 2 subjects were excluded

^4^ Due to technical difficulties, the scans of 3 subjects were excluded

^5^ Subjects were diagnosed with type I alcoholism

^6^ Due to technical difficulties, the scans of 2 subjects were excluded and for 1 subject only the right amygdala could be traced

^7^ Due to technical difficulties, the scan of 1 subject was excluded

^8^ While it is possible that some subjects were acquitted, it is reasonable to assume that most were convicted given the high level of correspondence between arrest and conviction rates for homicide (e.g. Forst, 2002).

**Table S2.** Processing characteristics of voxel-based morphometry (VBM) studies (*N* = 6) included in the Anatomical Likelihood Estimation (ALE) meta-analysis.

Reference Software Modulated Coordinate system Smoothing kernel Thresholding criteria

Bertsch et al. (2013) SPM8 Yes MNI 8 mm FWHM *p*_uncorrected_ < 0.005, minimum cluster size of 67.5 mm^3^

Bobes et al. (2013) SPM5 No MNI 8 mm FWHM *p*_uncorrected_ < 0.005

Contreras-Rodríguez et al. (2014) SPM8 Yes MNI 8 mm FWHM Minimum cluster size of 1000 mm^3^ calculated with 1000

Monte Carlo simulations, satisfying *p*_FWER_ < 0.05

Gregory et al. (2012) SPM5 Yes MNI 8 mm FWHM *z* > 2.7, cluster-corrected at *p* < 0.05 using GRFT

Schiffer et al. (2011) SPM5 Yes MNI 12 mm FWHM *p*_FDR_ < 0.05, spatial extent threshold based on GRFT

Tiihonen et al. (2008) SPM2 ns MNI 12 mm FWHM *p*_FDR_ < 0.05

FDR = false discovery rate; FWER = family-wise error rate; FWHM = full width at half maximum; GRFT = Gaussian random field theory; mm = millimeter; MNI = Montreal Neurological Institute; ns = not specified; SPM = statistical parametric mapping


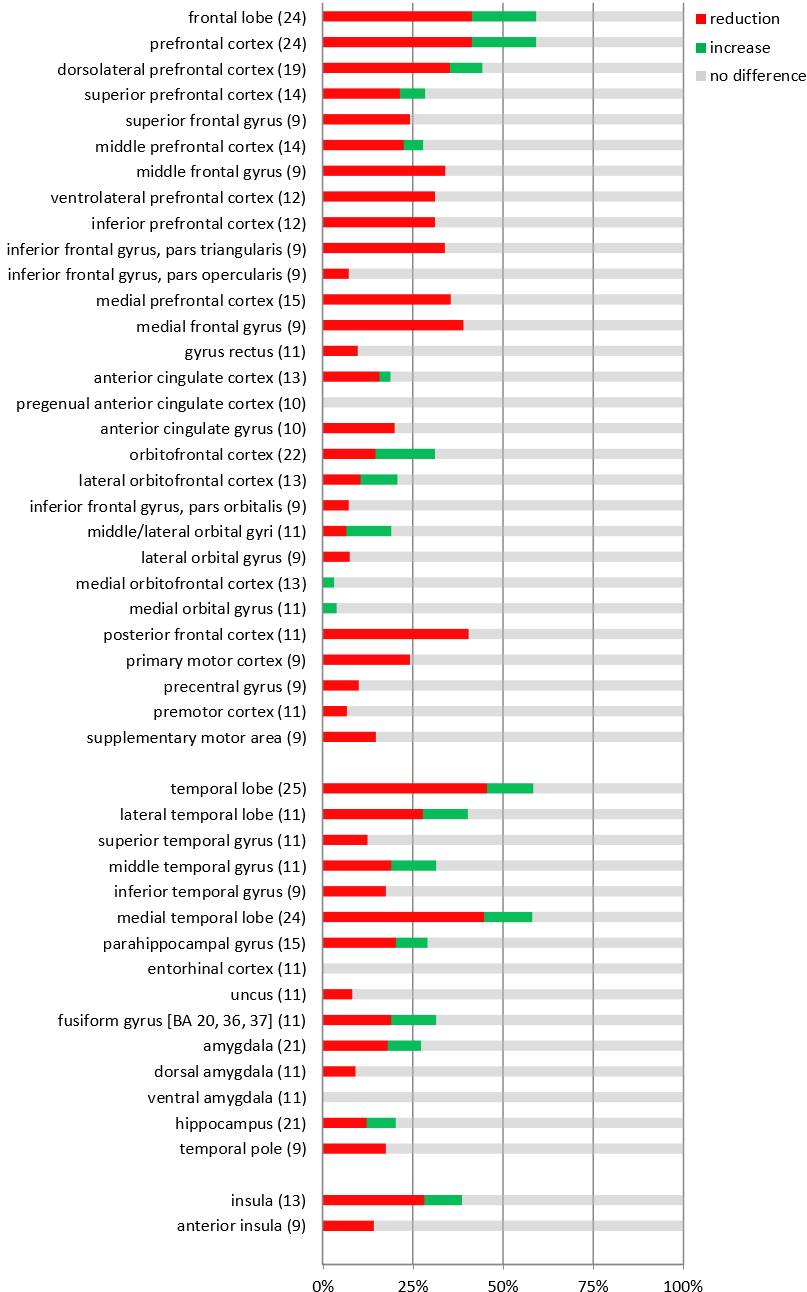


**Figure S2**. Percentages of neuroimaging experiments indicating whether interpersonal violence was associated with a reduction, increase or no difference in grey matter (GM) volume in discrete brain regions. Experiments were weighted by sample size. For each region, the total number of experiments is given between parentheses. BA = Brodmann Area.


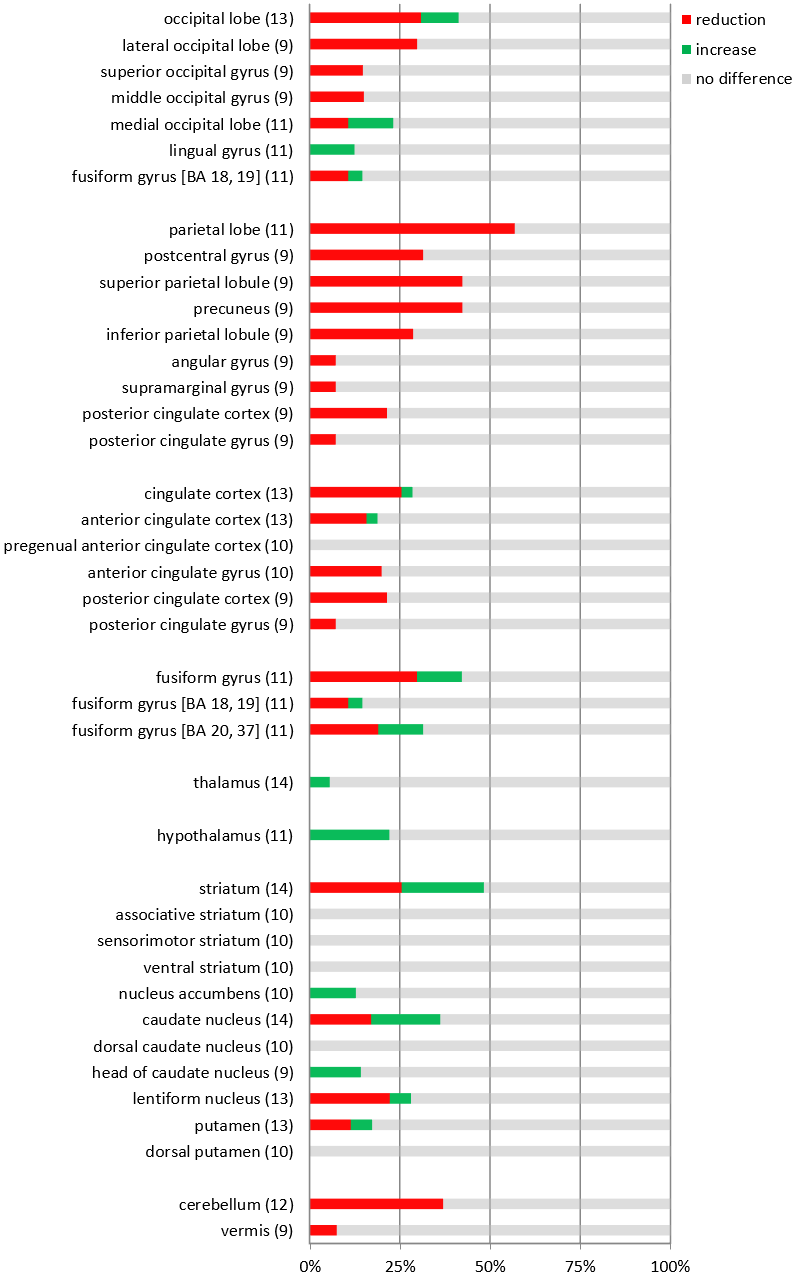


**Figure S2.** (continued)


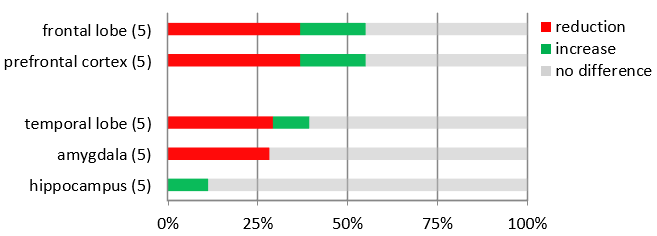


**Figure S3.** Percentages of neuroimaging experiments indicating whether reactive violence was associated with a reduction, increase or no difference in grey matter (GM) volume in discrete brain regions. Experiments were weighted by sample size. For each region, the total number of experiments is given between parentheses.


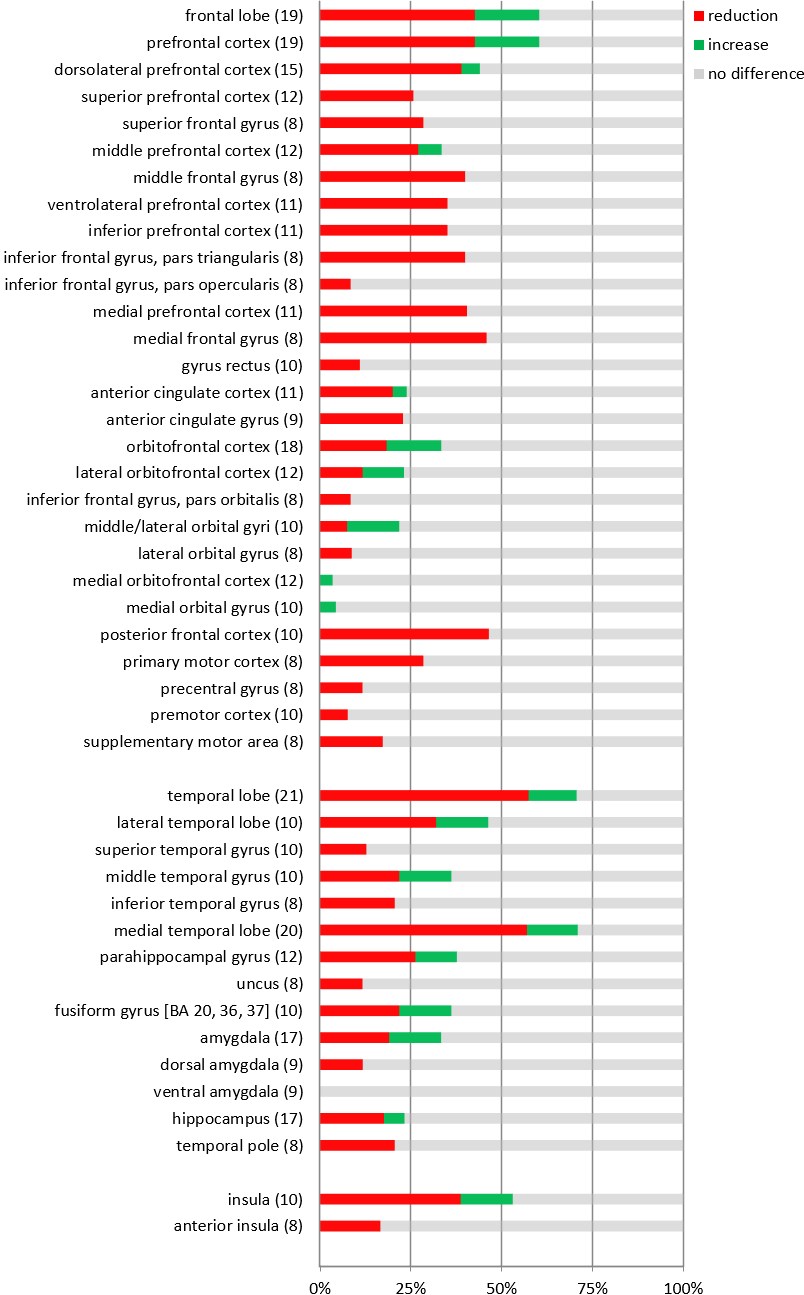


**Figure S4**. Percentages of neuroimaging experiments indicating whether interpersonal violence was associated with a reduction, increase or no difference in grey matter (GM) volume in discrete brain regions, excluding experiments in which reactive violence was used as outcome. Experiments were weighted by sample size. For each region, the total number of experiments is given between parentheses. BA = Brodmann Area.


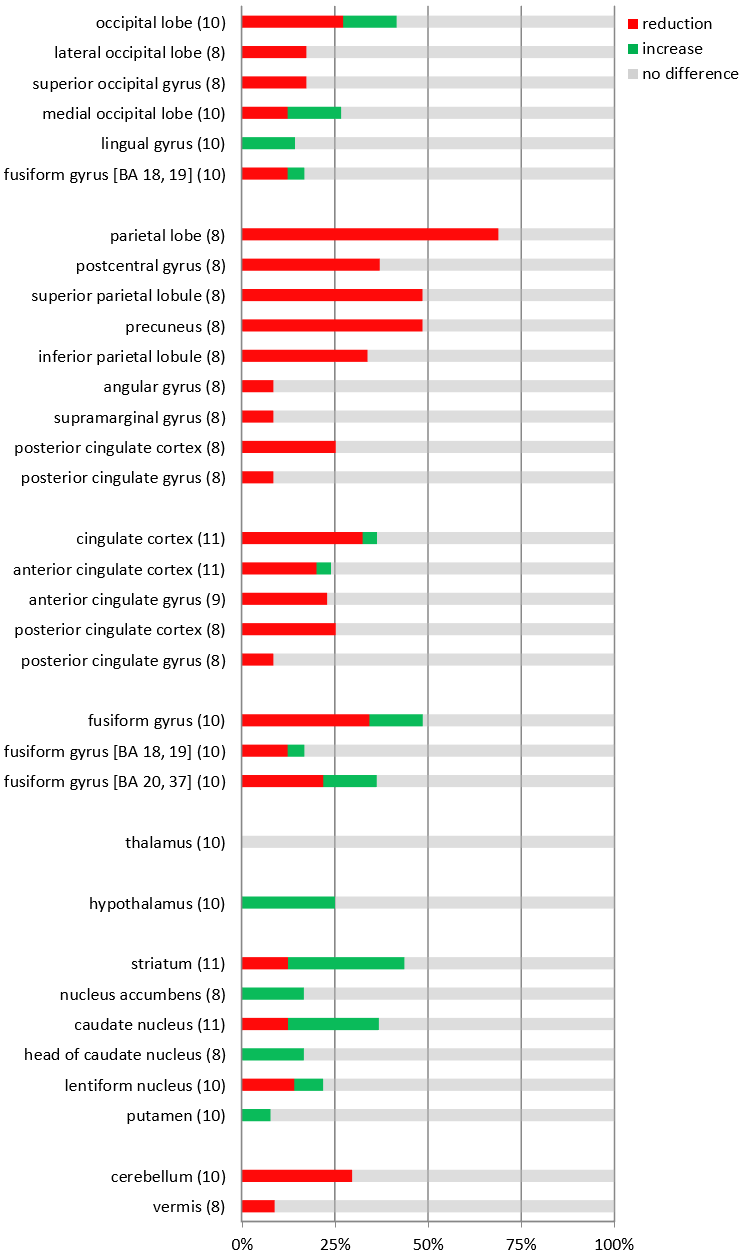


**Figure S4.** (continued)


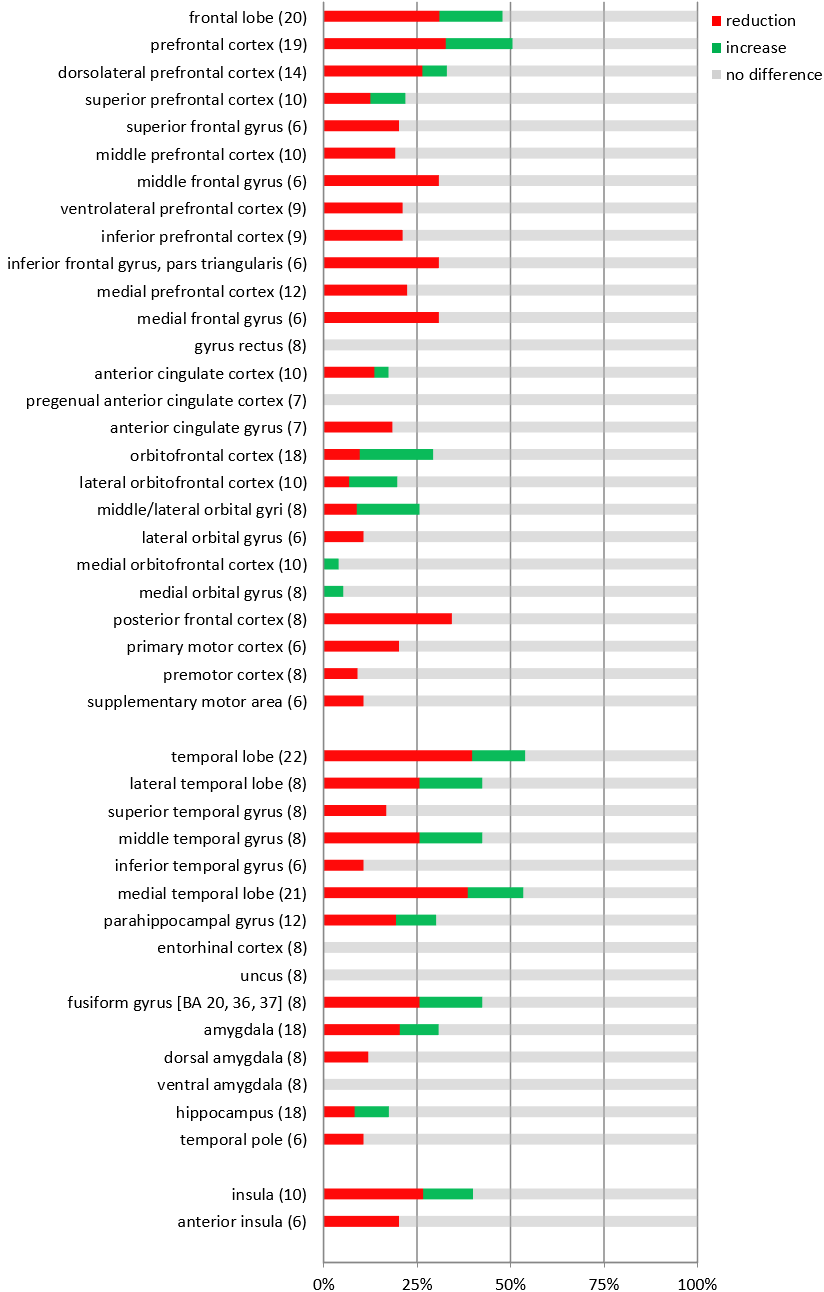


**Figure S5**. Percentages of neuroimaging experiments indicating whether interpersonal violence was associated with a reduction, increase or no difference in grey matter (GM) volume in discrete brain regions, excluding experiments with samples that consisted of subjects diagnosed with psychopathy. Experiments were weighted by sample size. For each region, the total number of experiments is given between parentheses. BA = Brodmann Area.


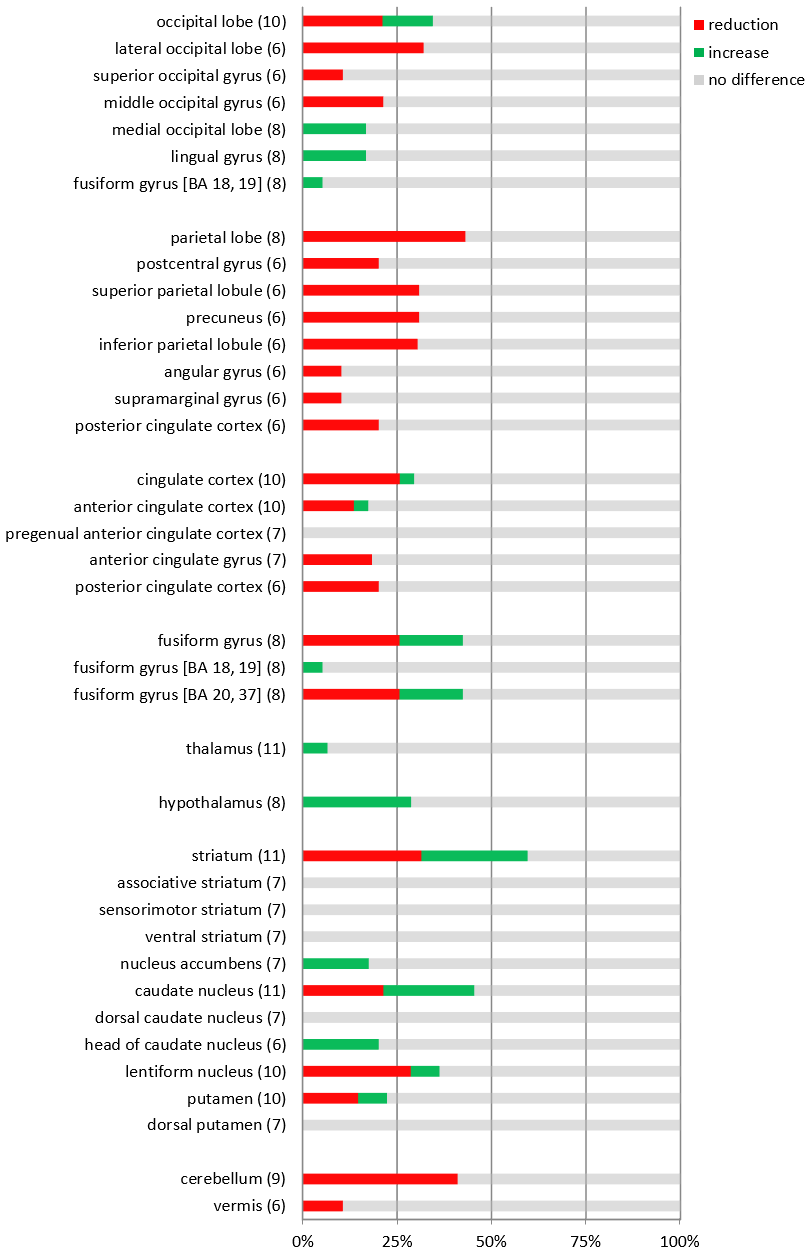


**Figure S5.** (continued)

**References**

[American Psychiatric Association, 1987](http://ezproxy-prd.bodleian.ox.ac.uk:2054/science/article/pii/019188699390156W#BIB1). Diagnostic and Statistical Manual of Mental Disorders, 3rd ed., rev. Author, Washington, DC.

American Psychiatric Association, 1994. Diagnostic and Statistical Manual of Mental Disorders, 4th ed. Author, Washington, DC.

American Psychiatric Association, 2000. Diagnostic and Statistical Manual of Mental Disorders, 4th ed., text rev. Author, Washington, DC.

Barkataki, I., Kumari, V., Das, M., Taylor, P., Sharma, T., 2006. Volumetric structural brain abnormalities in men with schizophrenia or antisocial personality disorder. Behav. Brain Res. 169, 239–247.

Bertsch, K., Grothe, M., Prehn, K., Vohs, K., Berger, C., Hauenstein, K., et al., 2013. Brain volumes differ between diagnostic groups of violent criminal offenders. Eur. Arch. Psychiatry Clin. Neurosci. 263, 593–606.

Blackburn, R., 1982. The special hospital assessment of personality and socialisation. Unpublished manuscript, Ashworth Hospital.

Bobes, M.A., Ostrosky, F., Diaz, K., Romero, C., Borja, K., Santos, Y., Valdés-Sosa, M., 2013. Linkage of functional and structural anomalies in the left amygdala of reactive-aggressive men. Soc. Cogn. Affect. Neurosci. 8, 928–936.

Brown, G.L., Goodwin, F.K., Ballenger, J.C., Goyer, P.F., Major, L.F., 1979. Aggression in humans correlates with cerebrospinal ﬂuid amine metabolites. Psychiatry Res. 1, 131–139.

Chinese Society of Psychiatry, 2001. The Chinese Classification and Diagnostic Criteria of Mental Disorders, 3rd ed. Shandong Science and Technology Press, Jinan, China.

Cloninger, C.R., 1987a. Neurogenetic adaptive mechanisms in alcoholism. Science 236, 410–416.

Cloninger, C.R., 1987b. A systematic method for clinical description and classification of personality variants: a proposal. Arch. Gen. Psychiatry 44, 573–588.

Cloninger, C.R., Bohman, M., Sigvardsson, S., 1981. Inheritance of alcohol abuse: cross-fostering analysis of adopted men. Arch. Gen. Psychiatry 38, 861–868.

Coccaro, E.F., Berman, M.E., Kavoussi, R.J., 1997. Assessment of life history of aggression: development and psychometric characteristics. Psychiatry Res. 73, 147–157.

Coccaro, E.F., Kavoussi, R.J., Berman, M., Lish, J., 1998. Intermittent explosive disorder-revised: development, reliability, and validity of research criteria. Compr. Psychiatry 39, 368–376.

Contreras-Rodríguez, O., Pujol, J., Batalla, I., Harrison, B.J., Soriano-Mas, C., Deus, J., et al., 2014. Functional connectivity bias in the prefrontal cortex of psychopaths. Biol. Psychiatry 78, 647–655.

Dolan, M.C., Deakin, J.F.W., Roberts, N., Anderson, I.M., 2002a. Quantitative frontal and temporal structural MRI studies in personality-disordered offenders and control subjects. Psychiatry Res. Neuroimaging 116, 133–149.

Dolan, M., Deakin, W.J.F., Roberts, N., Anderson, I., 2002b. Serotonergic and cognitive impairment in impulsive aggressive personality disordered offenders: are there implications for treatment? Psychol. Med. 32, 105–117.

Elliott, D.S., Huizinga, D., Ageton, S.S., 1985. Explaining Delinquency and Drug Use. Sage, Thousand Oaks, CA.

Frankle, W.G., Lombardo, I., New, A.S., Goodman, M., Talbot, P.S., Huang, Y., et al., 2005. Brain serotonin transporter distribution in subjects with impulsive aggressivity: a positron emission study with [11C]McN 5652. Am. J. Psychiatry 162, 915–923.

Forst, B., 2002. Charge attrition. In: Levinson, D. (Ed.), Encyclopedia of Crime and Punishment. Sage, Thousand Oaks, CA.

Gansler, D.A., Lee, A.K.W., Emerton, B.C., D'Amato, C., Bhadelia, R., Jerram, M., Fulwile, C., 2011. Prefrontal regional correlates of self-control in male psychiatric patients: impulsivity facets and aggression. Psychiatry Res. Neuroimaging 191, 16–23.

Gansler, D.A., McLaughlin, N.C.R., Iguchi, L., Jerram, M., Moore, D.W., Bhadelia, R., Fulwiler, C.A., 2009. Multivariate approach to aggression and the orbital frontal cortex in psychiatric patients. Psychiatry Res. Neuroimaging 171, 145–154.

Gilliam, M.C., 2014. Exposure to maternal depression during childhood and adolescence and problem behavior in early adulthood: a possible mediating role for brain structure (Master’s thesis, University of Pittsburgh). Retrieved from <http://d-scholarship.pitt.edu/19887>.

Gilliam, M., Forbes, E.E., Gianaros, P.J., Erickson, E.I., Brennan, L.M., Shaw, D.S., 2014. Maternal depression in childhood and aggression in young adulthood: evidence for mediation by offspring amygdala-hippocampal volume ratio. J. Child Psychol. Psychiatry 56, 1083–1091.

Gopal, A., Clark, E., Allgair, A., D’Amato, C., Furman, M., Gansler, D.A., Fulwiler, C., 2013. Dorsal/ventral parcellation of the amygdala: relevance to impulsivity and aggression. Psychiatry Res. Neuroimaging 211, 24–30.

Gregory, S., Ffytche, D., Simmons, A., Kumari, V., Howard, M., Hodgins, S., Blackwood, N., 2012. The antisocial brain: psychopathy matters: a structural MRI investigation of antisocial male violent offenders. Arch. Gen. Psychiatry 69, 962–972.

Gunn, J., Robertson, G., 1976. Drawing a criminal profile. Br. J. Criminol. 16, 156–160.

Gunderson, J.G., Kolb, J.E., Austin, V., 1981. The diagnostic interview for borderlines. Am. J. Psychiatry 138, 896–903.

Hare, R.D., 2003. The Hare Psychopathy Checklist-Revised. Multi Health Systems, Toronto, Canada.

Hoffstaedter, F., Grefkes, C., Caspers, S., Roski, C., Palomero-Gallagher, N., Laird, A.R., et al., 2014. The role of anterior midcingulate cortex in cognitive motor control: evidence from functional connectivity analyses. Hum. Brain Mapp. 35, 2741–2753.

Hoptman, M.J., Volavka, J., Czobor, P., Gerig, G., Chakos, M., Blocher, J., et al., 2006. Aggression and quantitative MRI measures of caudate in patients with chronic schizophrenia or schizoaffective disorder. J. Neuropsychiatry Clin. Neurosci. 18, 509–515.

Hoptman, M.J., Volavka, J., Weiss, E.M., Czobor, P., Szeszko, P.R., Gerig, G., et al., 2005. Quantitative MRI measures of orbitofrontal cortex in patients with chronic schizophrenia or schizoaffective disorder. Psychiatry Res. Neuroimaging 140, 133–145.

Kay, S.R., Wolkenfeld, F., Murrill, L.M., 1988. Profiles of aggression among psychiatric patients: nature and prevalence. J. Nerv. Ment. Dis. 176, 539–546.

Kiernan, J.A., 2012. Anatomy of the temporal lobe. Epilepsy Res. Treat. 2, 1–12.

Kozslovskiy, S.A., Vartanov, A.V., Nikonova, E.Y., Pyasik, M.M., Velichkovsky, B.M., 2012. The cingulate cortex and human memory processes. Psychol. Russia 6, 231–243.

Kumari, V., Barkataki, I., Goswami, S., Flora, S., Das, M., Taylor, P., 2009. Dysfunctional, but not functional, impulsivity is associated with a history of seriously violent behaviour and reduced orbitofrontal and hippocampal volumes in schizophrenia. Psychiatry Res. Neuroimaging 173, 39–44.

Kumari, V., Gudjonsson, G.H., Raghuvanshi, S., Barkataki, I., Taylor, P., Sumich, A., et al., 2013. Reduced thalamic volume in men with antisocial personality disorder or schizophrenia and a history of serious violence and childhood abuse. Eur. Psychiatry 28, 225–234.

Kumari, V., Uddin, S., Premkumar, P., Young, S., Gudjonsson, G.H., Raghuvanshi, S., et al., 2014. Lower anterior cingulate volume in seriously violent men with antisocial personality disorder or schizophrenia and a history of childhood abuse. Aust. N. Z. J. Psychiatry 48, 153–161.

Laakso, M.P., Gunning-Dixon, F., Vaurio, O., Repo-Tiihonen, E., Soininen, H., Tiihonen, J., 2002. Prefrontal volumes in habitually violent subjects with antisocial personality disorder and type 2 alcoholism. Psychiatry Res. Neuroimaging 114, 95–102.

Laakso, M.P., Vaurio, O., Savolainen, L., Repo, E., Soininen, H., Aronen, H.J., Tiihonen, J., 2000. A volumetric MRI study of the hippocampus in type 1 and 2 alcoholism. Behav. Brain Res. 109, 177–186.

Matthies, S., Rüsch, N., Weber, M., Lieb, K., Philipsen, A., Tuescher, O., et al., 2012. Small amygdala – high aggression? The role of the amygdala in modulating aggression in healthy subjects. World J. Biol. Psychiatry 13, 75–81.

McCloskey, M.S, Berman, M.E., Noblett, K.L., Coccaro, E.F., 2006. Intermittent explosive disorder-integrated research diagnostic criteria: convergent and discriminant validity. J. Psychiatr. Res. 40, 231–242.

Moher, D., Liberati, A., Tetzlaff, J., Altman, D.G., The PRISMA Group, 2009. Preferred reporting items for systematic reviews and meta-analyses: the PRISMA statement. PLoS Med. 6, 1–6.

New, A.S., Hazlett, E.A., Buchsbaum, M.S., Goodman, M., Mitelman, S.A., Newmark, R., et al., 2007. Amygdala-prefrontal disconnection in borderline personality disorder. Neuropsychopharmacology 32, 1629–1640.

Nolte, J., 2009. The Human Brain: an Introduction to Its Functional Anatomy, 6th ed. Moby Elsevier, Philadelphia, PA.

Pardini, D.A., Raine, A., Erickson, K., Loeber, R., 2014. Lower amygdala volume in men is associated with childhood aggression, early psychopathic traits, and future violence. Biol. Psychiatry 75, 73–80.

Puri, B.K., Counsell, S.J., Saeed, N., Bustos, M.G., Treasaden, I.H., Bydder, G.M., 2008. Regional grey matter volumetric changes in forensic schizophrenia patients: an MRI study comparing the brain structure of patients who have seriously and violently offended with that of patients who have not. BMC Psychiatry 8, 1–6.

Rademacher, J., Galaburda, A.M., Kennedy, D.N., Filipek, P.A., Caviness, V.S., 1992. Human cerebral cortex: localization, parcellation, and morphometry with magnetic resonance imaging. J. Cognitive Neurosci. 4, 352–374.

Radua, J., Canales-Rodríguez, E.J., Pomarol-Clotet, E., Salvador, R., 2014. Validity of modulation and optimal settings for advanced voxel-based morphometry. Neuroimage 86, 81–90.

Raine, A., Dodge, K., Loeber, R., Gatzke-Kopp, L., Lynam, D., Reynolds, C., et al., 2006. The reactive-proactive aggression questionnaire: differential correlates of reactive and proactive aggression in adolescent boys. Aggress. Behav. 32, 159–171.

Rorden, C., Karnath, H.-O., Bonilha, L., 2007. Improving lesion-symptom mapping. J. Cognitive Neurosci. 19, 1081–1088.

Rosell, D.R., Thompson, J.L., Slifstein, M., Xu, X., Frankle, W.G., New, A.S., et al., 2010. Increased serotonin 2A receptor availability in the orbitofrontal cortex of physically aggressive personality disordered patients. Biol. Psychiatry 67, 1154–1162.

Schiffer, B., Leygraf, N., Müller, B.W., Scherbaum, N., Forsting, M., Wiltfang, J., et al., 2013. Structural brain alterations associated with schizophrenia preceded by conduct disorder: a common and distinct subtype of schizophrenia? Schizophr. Bull. 39, 1115–1128.

Schiffer, B., Müller, B.W., Scherbaum, N., Hodgins, S., Forsting, S., Wiltfang, J., et al., 2011. Disentangling structural brain alterations associated with violent behavior from those associated with substance use disorders. Arch. Gen. Psychiatry 68, 1039–1049.

Soloff, P., White, R., Diwadkar, V.A., 2014. Impulsivity, aggression and brain structure in high and low lethality suicide attempters with borderline personality disorder. Psychiatry Res. Neuroimaging 222, 131–139.

Spoletini, I., Piras, F., Fagioli, S., Rubino, I.A., Martinotti, G., Siracusano, A., et al., 2011. Suicidal attempts and increased right amygdala volume in schizophrenia. Schizophr. Res. 125, 30–40.

Stanford, M.S., Houston, R.J., Mathias, C.W., Villemarette-Pittman, N.R., Helfritz, L.E., Conklin, S.M., 2003. Characterizing aggressive behavior. Assessment 10, 183–190.

Straus, M.A., 1979. Measuring intrafamily conflict and violence: the conflict tactics scales. J. Marriage Fam. 41, 75–88.

Tiihonen, J., Rossi, R., Laakso, M.P., Hodgins, S., Testa, C., Perez, J., et al., 2008. Brain anatomy of persistent violent offenders: more rather than less. Psychiatry Res. Neuroimaging 163, 201–212.

Tzourio-Mazoyer, N., Landeau, B., Papathanassiou, D., Crivello, F., Etard, O., Delcroix, N., et al., 2002. Automated anatomical labeling of activations in SPM using a macroscopic anatomical parcellation of the MNI MRI single-subject brain. Neuroimage 15, 273–289.

Van de Giessen, E., Rosell, D.R., Thompson, J.L., Xu, X., Girgis, R.R., Ehrlich, Y., et al., 2014. Serotonin transporter availability in impulsive aggressive personality disordered patients: a PET study with [11C]DASB. J. Psychiatr. Res. 58, 147–154.

World Health Organization, 1992. The ICD-10 Classification of Mental and Behavioral Disorders: Clinical Descriptions and Diagnostic Guidelines. World Health Organization, Geneva, Switzerland.

Yang, Y., Raine, A., 2009. Prefrontal structural and functional brain imaging findings in antisocial, violent, and psychopathic individuals: a meta-analysis. Psychiatry Res. Neuroimaging 174, 81–88.

Yang, Y., Raine, A., Han, C.-B., Schug, R.A., Toga, A.W., Narr, K.L., 2010. Reduced hippocampal and parahippocampal volumes in murderers with schizophrenia. Psychiatry Res. Neuroimaging 182, 9–13.

Yudofsky, S.C., Silver, J.M., Jackson, W., Endicott, J., Williams, D., 1986. The overt aggression scale for the objective rating of verbal and physical aggression. Am. J. Psychiatry 143, 35–39.

Zanarini, M., Gunderson, J.G., Frankenburg, F.R., Chauncey, D.L., 1989. The revised diagnostic interview for borderlines: discriminating BPD from other Axis II disorders. J. Pers. Disord. 3, 10–18.

Zetzsche, T., Preuss, U.W., Bondy, B., Frodl, T., Zill, P., Schmitt, G., et al., 2008. 5-HT1A receptor gene C-1019 G polymorphism and amygdala volume in borderline personality disorder. Genes Brain Behav. 7, 306–313.

Zetzsche, T., Preuss, U.W., Frodl, T., Schmitt, G., Seifert, D., Münchhausen, E., et al., 2007. Hippocampal volume reduction and history of aggressive behaviour in patients with borderline personality disorder. Psychiatry Res. Neuroimaging 154, 157–170.

Zhang, L., Kerich, M., Schwandt, M.L., Rawlings, R.R., McKellar, J.D., Momenan, R., et al., 2013. Smaller right amygdala in Caucasian alcohol-dependent male patients with a history of intimate partner violence: a volumetric imaging study. Addict. Biol. 18, 537–547.
